# Synthesis and performance study of cationic surfactants containing different quantities of hydroxyl

**DOI:** 10.1039/d5ra00182j

**Published:** 2025-03-07

**Authors:** Shuai Gao, Yajie Jiang, Lu Zhang, Jun Li, Yakui Wang, Zhifei Wang, Yongsheng Lan, Tao Geng

**Affiliations:** a China Research Institute of Daily Chemical Industry Taiyuan 030001 Shanxi China; b Shanxi Key Laboratory of Functional Surfactants Taiyuan 030001 Shanxi China ridcigt@163.com

## Abstract

Three hydroxyl-containing cationic surfactants are synthesized and characterized by FTIR and ^1^HNMR, and their thermal stability is tested and analyzed. The surface activity, adsorption and aggregation behavior of the synthesized target product were investigated by testing its contact angle, static surface tension and dynamic surface tension. As the number of hydroxyl groups increases, the maximum adsorption amount (*Γ*_max_) of molecules at the interface gradually decreases, and the minimum area occupied by each molecule (*A*_min_) increases, suggesting that the introduction of hydroxyl groups weakens the interfacial accumulation ability between molecules and increases the hydrophilicity. In addition, cationic surfactants with different hydroxyl groups show clear differences in the performance test. The DMAE exhibits excellent foaming ability and salt tolerance, while TMA offers the best alkali resistance. In comparison, MDAE provides greater advantages in terms of dynamic surface tension and wetting time, while also demonstrating strong anti-static properties. In short, the TMA is suitable for scenarios with high requirements for alkali resistance and static adsorption performance. The comprehensive performance of the DMAE is the best, suitable for scenarios with high requirements of salt resistance, foam performance and anti-static performance. The MDAE possesses outstanding dynamic performance, making it suitable for applications requiring rapid wetting and dynamic surface adaptation.

## Abstract 2

Three hydroxyl-containing cationic surfactants are synthesized and characterized by FTIR and ^1^HNMR, and their thermal stability is tested and analyzed.

## Graphical abstract

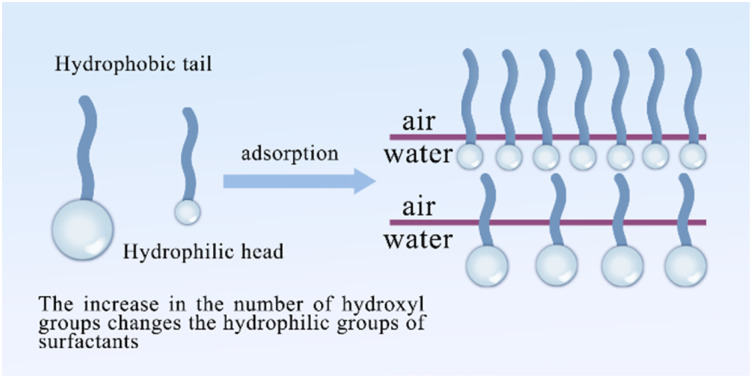

## 1. Introduction

Surfactants are a widely used class of chemical substances that can be used to alter the properties of surfaces or interfaces. Their main functions include reducing surface tension, promoting wetting, emulsifying, solubilizing and dispersing.^1^ Surfactants consist of both hydrophilic and hydrophobic groups and have many unique properties such as: emulsification, solubilization and wetting properties.^2–5^ Due to these properties, surfactants are widely used in many areas and play an important role, particularly in everyday life and industrial production.^6,7^

The hydrophilic head and hydrophobic tail of a surfactant play a crucial role in determining its physicochemical properties. Modifying existing surfactants by introducing certain functional groups is one of the approaches to synthesizing desired surfactants. Hydroxyl-containing surfactants not only lower the critical micelle concentration (CMC) and equilibrium surface tension but also enhance solubility and impart other desirable properties.^7–9^ In the utilization of iron ore, introducing hydroxyl groups into cationic surfactants can enhance the collector's surface activity and selective adsorption on the desired mineral surface. Liu designed a hydroxyl-containing polyamine cationic surfactant to improve the separation efficiency of the reverse flotation system for hematite ore.^10^ Zhang's research revealed that substituting methyl groups with hydroxyethyl groups in the surfactant head leads to an increase in micellization volume changes, along with a reduction in critical micelle concentration (CMC), counterion binding degree, and Δ*G*° values. However, the introduction of hydroxyethyl had little effect on the aggregation number of these surfactants in aqueous solutions. It was also found that the amic value increases with the incorporation of hydroxyethyl groups.^11^ C22-tail surfactants have attracted significant attention due to their ability to self-assemble into worm-like micelles and exhibit viscoelastic behavior similar to polymer solutions. However, they typically contain easily degradable double bonds.^12^ Zhang was the first to report the worm-like micelles and solution properties of the saturated C22-tail carboxybetaine surfactant DDCB. It possesses an intermediate Krafft temperature (TK), which favors solubility under *in situ* application conditions. The long hydrophobic tail of DDCB contributes to a low critical micelle concentration (CMC) and a small molecular area per molecule, facilitating the formation of long cylindrical worm-like micelles and significantly enhancing the viscoelasticity of the solution.^13^ It is obvious that the type and number of compound groups on surfactants have a significant impact on their performance. However, current research focuses mainly on the influence of the position of linking groups on their characteristics, while studies on the influence of their number remain scarce.

**Fig. 1:**
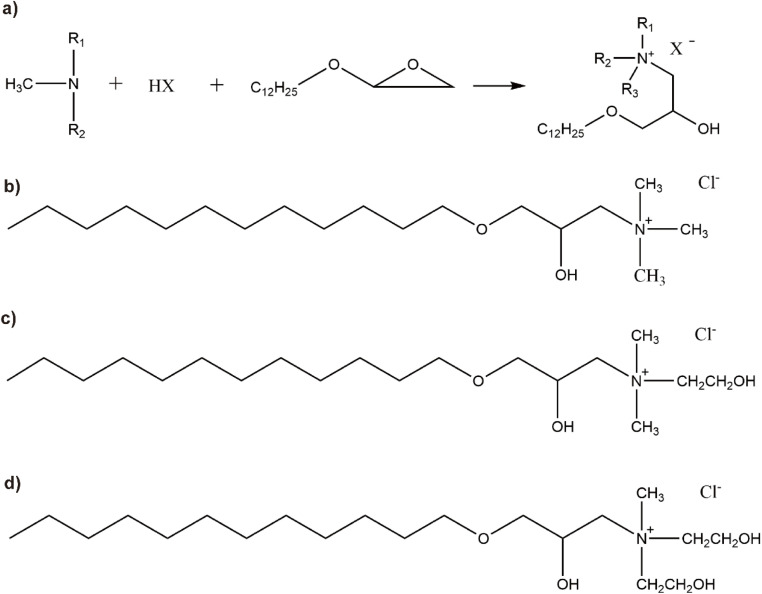
(a) The reaction process for synthesizing hydroxyl-containing cationic surfactants (*R*_1_, *R*_2,_ and *R*_3_ are CH_3_, CH_3_CH_2_, CH_3_CH_2_CH_2_, or CH_2_CH_2_OH; *X* represents Cl^−^, Br^−^, HCOO^−^, CH_3_COO^−^, or CH_3_CH_2_COO^−^). (b)–(d)The structural formulas of the synthesized products (TMA, DMAE, MDAE).

In this research, a variety of cationic surfactants featuring different quantities of hydroxyl groups were prepared using a straightforward two-step synthesis method. The structures of the synthesized surfactants were characterized using FTIR, ^1^HNMR, and elemental analysis, with Fig. 1a illustrating the synthetic route of the cationic surfactants. The effects of different hydroxyl group numbers on surfactant performance were compared by testing surface tension, contact angle, foam properties, antistatic ability, alkali resistance, salt, resistance and thermal stability.

## 2. Experimental section

### 2.1 Materials

Trimethylamine hydrochloride (white granules, 98%) was purchased from Shanghai Kelin Biochemical Technology Co, Ltd., and *N*,*N*-dimethylethanolamine and *N*-methyldiethanolaMine were purchased from Tianjin Damao Chemical Reagent Factory. *n*-Dodecyl glycidyl ether is purchased from Shandong Foster Oil Technology Co., Ltd., ethanol and ethyl acetate are purchased from Tianjin Comeo Chemical Reagent Co., Ltd., and hydrochloric acid (aqueous solution, 37.5%) is purchased from Sinopharm Group Chemical Reagent Co., Ltd.

### 2.2 Synthesis

First, methyl diethanolamine (MDEA) and dimethyl ethanolamine (DMEA) are separately neutralized with an acid. After the neutralization reaction, they are reacted with dodecyl glycidyl ether to synthesize the target products. Trimethylamine hydrochloride (TMA) is directly reacted with dodecyl glycidyl ether to obtain the target products: *N*,*N*-bis(2-hydroxyethyl)-*N*-(2-methyl)-*N*-(3-dodecyloxy-2-hydroxy)ammonium chloride (MDAE), *N*-(2-hydroxyethyl)-*N*,*N*-bis(2-methyl)-*N*-(3-dodecyloxy-2-hydroxy)ammonium chloride (DMAE), and *N*,*N*,*N*-(2-methyl)-*N*-(3-dodecyloxy-2-hydroxy)ammonium chloride (TMA). The active ingredient content exceeds 97%. The reaction process and the structural formulas of the reaction products are shown in Fig. 1a and b.

### 2.3 Characterization

The product structure was characterized by FTIR, ^1^HNMR, and elemental analysis. Fourier transform infrared spectroscopy (FTIR, VERTEX 70) detects functional group information in samples by analyzing the absorption properties of molecules to infrared light. Proton nuclear magnetic resonance (^1^HNMR, DRX 300, Bruker) determines the structure of organic compounds by detecting the magnetic resonance signals of hydrogen nuclei in the sample.

### 2.4 Static surface tension

At a temperature of 25.0 ± 0.1 °C, the equilibrium surface tension was measured five times using the platinum ring method and the single-point method with a Sigma 700 tensiometer (Swedish Biolin Scientific). The average surface tension value was recorded. Prior to each test, the sample was prepared and allowed to stand for 24 hours to ensure experimental accuracy. Before each measurement, the platinum ring was washed with distilled water and heated with an alcohol lamp until it glowed red, after which measurements were taken. The surface tension of calibrated deionized water was 72.0 ± 0.2 mN m^−1^.^14^1
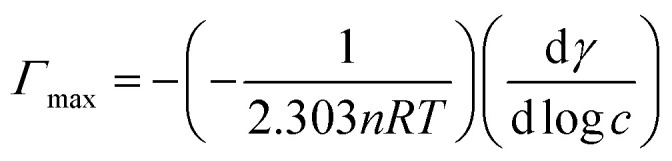
2
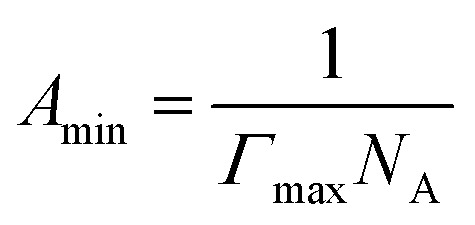


*T* represents the absolute temperature (298.15 K), *R* denotes the gas constant (8.314 J mol^−1^ K^−1^), and d*γ*/dlog *c* refers to the slope of the *γ*-log *c* curve below the critical micelle concentration (CMC). *n* is one constant determined by the number of species constituting the surfactant adsorbed at the interface. For linear cationic surfactants, *n* is equal to 2. *N*_A_ represents Avogadro's constant (6.022 × 10^23^).^15^

3 PC_20_ = −log *C*_20_

4
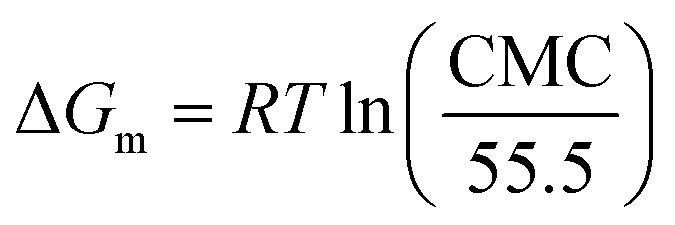
5
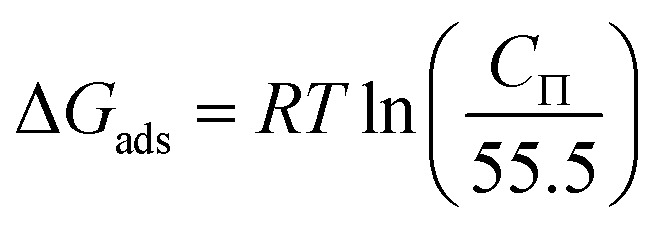


Here, *C*_Π_ represents the concentration of the surfactant in the aqueous phase under surface pressure.

### 2.5 Dynamic surface tension (DST)

The dynamic surface tension was tracked for 200 seconds using a BP100 bubble pressure tensiometer (KRÜSS, Germany) based on the bubble pressure method *γ*_0_.6
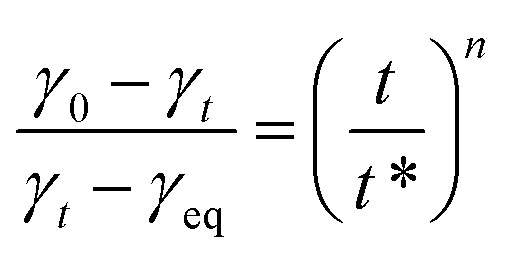
Here *γ*_eq_ is the equilibrium surface tension, *γ*_0_ is the surface tension of the solvent and *γ*_*t*_ is the surface tension of the solution at time *t*. t* and *n* are constants and eqn (7) can be used to calculate t* and *n*:^16^7
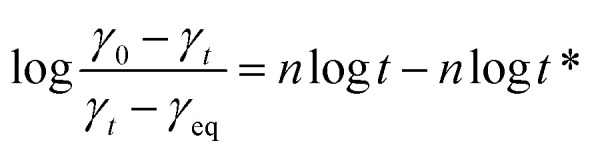
Here t* represents the time parameter when the surface tension drops to a certain level, *π*_*t*_ = *γ*_0_ − *γ*_*t*_ is the surface pressure at time *t* and *π*_eq_ = *γ*_0_ − *γ*_eq_ is the equilibrium surface pressure. log *K* = *π*_*t*_/*π*_eq_, a linear relationship was established by treating log *t* as an independent variable and log *K* as a dependent variable.8
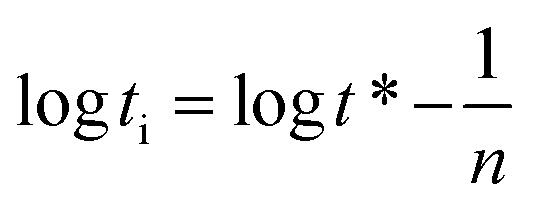
9
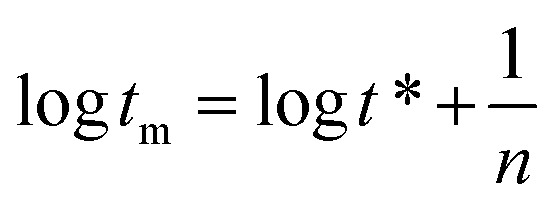
In the formulas, *t*_i_ is defined as the time marking the end of the induction region, and *t*_m_ is defined as the time marking the beginning of the mid-equilibrium region. *R*_1/2_ is defined as the rate of surface tension reduction during the rapid decline stage at time t*:^17^10
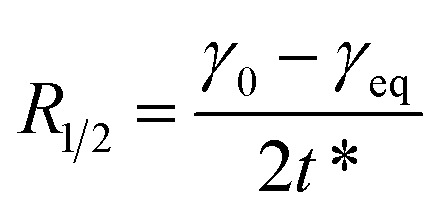
11
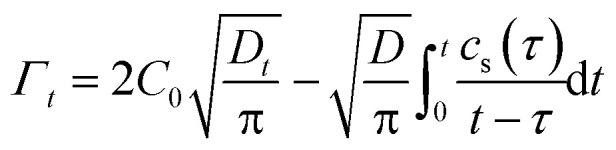
where *C*_0_ and *c*_s_ are the mass and surface concentrations, *τ* is the integration variable, *D* is the diffusion coefficient and *Γ*_*t*_ is the surface adsorption at time *t*.12
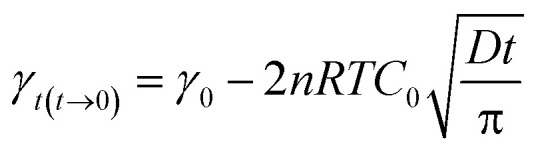
where *γ*_0_ is the surface tension of water, *γ*_*t*_ is the surface tension at time *t*, and for ionic surfactants, *n* is equal to 2.13
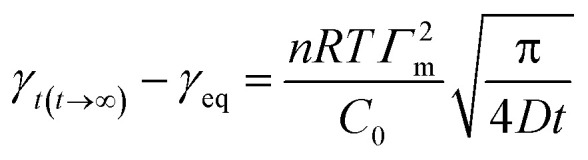


Here, *Γ*_eq_ represents the equilibrium surface excess concentration, while *γ*_eq_ denotes the equilibrium surface tension as *t* approaches infinity.

### 2.6 Contact angle test

The wettability of droplets on paraffin films was evaluated using a DSA25S drop shape analyzer (KRÜSS, Germany) through contact angle measurements. The final result was calculated as the average of five tests.

14 *γ*_gs_ = *γ*_ls_ + *γ*_gl_ cos*θ*

*γ*_gs_ is the free energy of the solid surface, which can be regarded as a constant under the same matrix. *γ*_ls_ and *γ*_gl_ are liquid–solid interface tension and gas–liquid surface tension respectively, and *θ* is the equilibrium contact angle of the droplet on the matrix.

### 2.7 Foamability and foam stability

Use FoamScan (FoamScan IT Concept, Teclis Co., France) to monitor foam performance through conductivity measurement and image analysis. To measure, inject 60 mL of surfactant solution into the glass sample cuvettes. Foaming involves introducing dry nitrogen into the liquid at a constant foaming rate of 150 mL min^−1^ until the foam formation time reaches 60 seconds and aeration stops. In this process, the CCD camera works with the cell size analysis (CSA) software to record and capture the image of the bubbles throughout the process.15
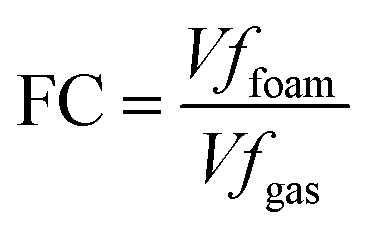
16
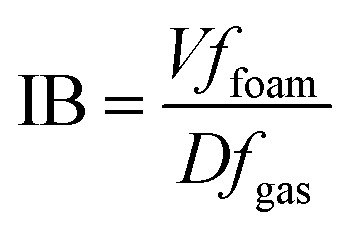
Here *V* represents the volume, *C* is the conductivity, the index *i* denotes the initial state (before the gas bubbles through the test solution) and *f* denotes the final state (at the end of the bubbling process).

### 2.8 Salt and alkali tolerance

For the salt and alkali resistance tests, a 10 g per L surfactant solution, a 200 g per L sodium chloride solution and a 200 g per L sodium hydroxide solution were prepared. Different amounts of sodium chloride and sodium hydroxide solutions were added to 10 mL of the surfactant solution to produce solutions with different salt and alkali concentrations. Each solution was subsequently heated to varying temperatures, and its transmittance was analyzed with a UV-vis spectrophotometer (Cary 60, USA). The surfactant's resistance to salts and alkalis was assessed once the solution's permeability achieved a level of 80%.

### 2.9 Wettability

The wetting performance of the surfactant was evaluated by measuring the wetting time of textiles at 25.0 ± 0.1 °C. The timing began at the moment the standard piece of canvas came into contact with the solution and continued until it was fully submerged and began to sink. The recorded time was used to assess the wetting performance of the product. The final result was calculated as the average of five measurements.

### 2.10 Emulsifying power

Using a cylinder with stopper, 40 mL of the oil phase (liquid paraffin or soybean oil) and 1 g L^−1^ of the aqueous phase (surfactant mixture) were added. The mixture was shaken vigorously five times per minute to ensure thorough mixing of the oil and water. The time required for 10 mL of water to separate from the emulsion was recorded. Each sample was tested five time and the average value was calculated.

### 2.11 Anti-static power

Polyester fabrics were cut into 100 mm × 100 mm squares and washed with a 1 g per L soap solution at 45 °C and then air dried. The fabric pieces were then immersed in 200 mL of a 10 g per L surfactant solution for 10 minutes, stirring occasionally with a glass rod. After treatment, the fabric samples were air dried and placed in a 45 °C oven to dry for 4 hours, ensuring that the samples did not touch each other. The surface resistance of fabric samples, measured both before and after treatment with the respective solutions, was assessed using a PC68 digital ultra-high resistance meter. The antistatic properties of five cationic surfactants with differing numbers of hydroxyl groups were assessed by calculating the logarithmic decrease in surface resistance. To ensure accuracy, each measurement was conducted five times, and the results were averaged.17
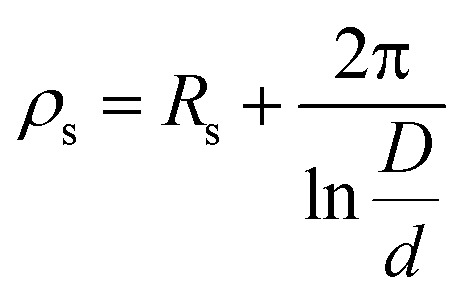


The inner diameters of the annular guard ring and the measuring electrode are denoted as *D* and *d*, respectively.

## 3. Results and discussion

### 3.1 Structural characterization

#### 3.1.1 FT-IR spectra

**Fig. 2:**
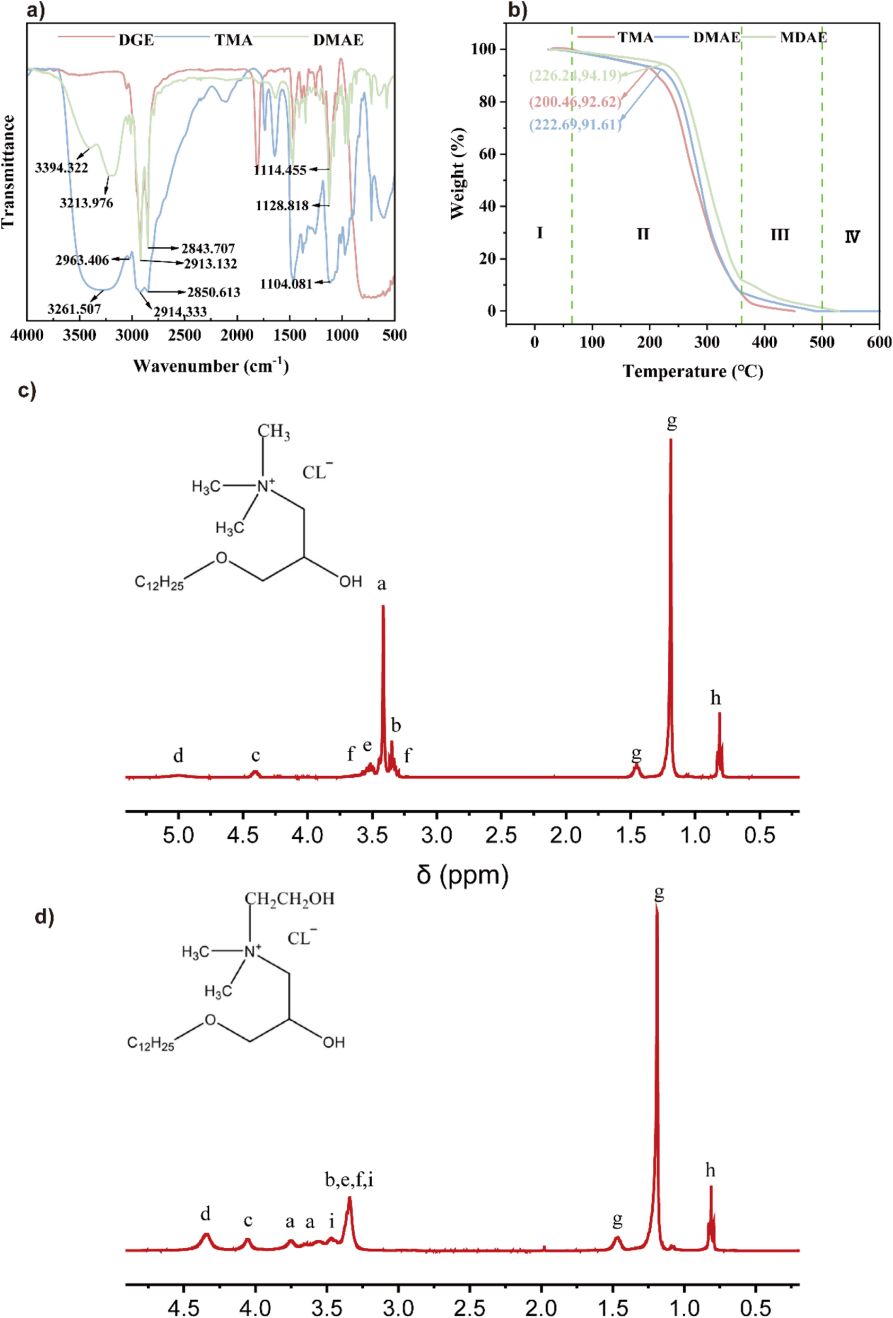
(a) FT-IR spectra, (b) and (c) ^1^HNMR spectrum, (d) TGA results.

Fourier transform infrared spectroscopy (FTIR, VERTEX 70) detects functional group information in samples by analyzing the absorption properties of molecules to infrared light. As illustrated in Fig. 2a, the peaks observed at 3200–3600 cm^−1^ confirm the presence of hydroxyl groups (OH) in the synthesized compounds. Similarly, the peaks in the 2800–3000 cm^−1^ range are characteristic of methyl groups (CH_3_), while those near 1100 cm^−1^ correspond to ether bonds (C–O–C) within the synthesized compounds.^18–21^ As shown in Fig. 2a, the molecular structures of the three substances are similar. DGE is the raw material, dodecyl glycidyl ether, and the changes in sample properties are attributed to the introduction of amines with different numbers of hydroxyl groups.

#### 3.1.2 ^1^HNMR spectra

The detailed results are shown in Table 1.

**Table 1:** ^1^HNMR spectrum: TMA (2c), MDAE (2d)^a^

Sample	*δ* (ppm)	Multiplicity	*J* (Hz)	Protons	Assignment
Fig. 2c	5.00	s	—	1H	–OH
	4.40	td	*J* = 8.6, 4.5	1H	–CH–
	3.62–3.54	m	—	1H	–CH_2_CH_2_O–
	3.51	dd	*J* = 9.6, 4.1	2H	–CHCH_2_O–
	3.41	s	—	9H	–N + (CH_3_)_3_
	3.35	t	*J* = 6.7	2H	–N + CH_2_
	3.32–3.25	M	—	1H	–CH_2_CH_2_O–
	1.45	P	*J* = 6.7	2H	–CH_2_CH_2_CH_2_–
	1.19	S	—	18H	–CH_2_(CH_2_)_9_CH3
	0.81	T	*J* = 6.7	3H	–(CH_2_)_9_CH_3_
Fig. 2d	4.56–4.22	m	—	2H	–OH
	4.16–3.99	m	—	1H	–CH–
	3.75	s	—	3H	–N + CH_3_
	3.71–3.53	m	—	3H	–N + CH_3_
	3.45	d	*J* = 13.3	2H	–N + CH_2_
	3.36	d	*J* = 14.2	8H	–CH_2_N + CH_2_–CHCH_2_OCH_2_–OCH_2_CH_2_–
	1.59–1.41	m	—	2H	–OCH_2_CH_2_–
	1.19	d	*J* = 3.9	18H	–CH_2_(CH_2_)_9_CH_3_
	0.81	t	*J* = 6.7	3H	–(CH_2_)_9_CH_3_

a Footnotes: s = singlet; d = doublet; t = triplet; m = multiplet; p = quartet; dd = doublet of doublets; td = triplet of doublets.

### 3.2 Thermal performance analysis

In this work, the thermal decomposition process of three synthesized surfactants was analyzed. As shown in Fig. 2b, stage 1 involves the decomposition of trace contaminants at room temperature. In stage 2, the synthesized samples decompose rapidly, resulting in a large drop in mass, with the temperature ranging between about 200 °C and 350 °C. The figure illustrates different initial decomposition temperatures for the surfactants: TMA > DMAE > MDAE. In the third stage, a slow decline is observed, with the mass ranking as TMA < DMAE < MDAE. This is likely attributable to the increased hydroxyl group count, which improves thermal stability. In the fourth stage, between 600 °C and 800 °C, minimal mass changes occur, indicating that the surfactants are almost completely decomposed.

### 3.3 Static surface tension

**Fig. 3:**
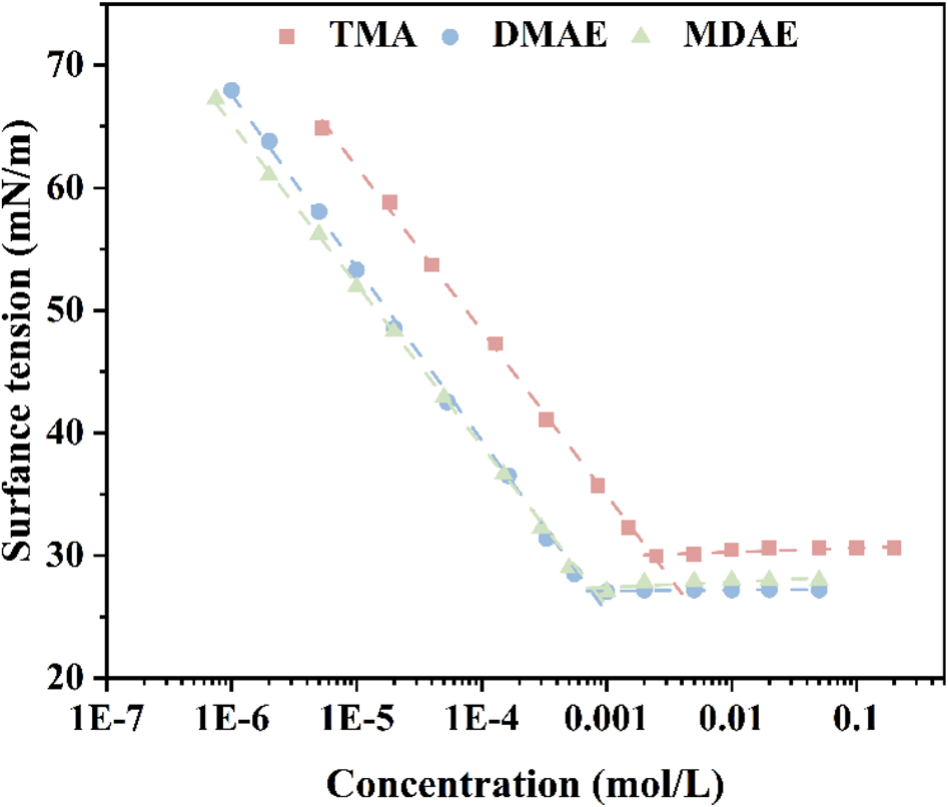
Surface tension of aqueous solutions of TMA, DMAE, and MDAE.

The surface tension measurements of TMA, DMAE, and MDAE are shown in Fig. 3. As the surfactant concentration increases, the surface tension gradually decreases, which is mainly related to the structure of the surfactant molecules and their adsorption behavior at the interface. When the surfactant molecules at the interface reach their maximum adsorption capacity, the molecular density at the interface tends to saturate and further increases in concentration can no longer significantly reduce the surface tension. At this point, the surface tension reaches its minimum value, referred to as *γ*CMC. The detailed results are shown in Table 2, the uncertainty value of the data in the table is 2.8%. The interfacial properties of surfactants (TMA, DMAE, MDAE) at the gas–liquid boundary were further evaluated through the Gibbs adsorption equation (eqn (18) and (19)). Comprehensive results are provided in Table 2.^22^18
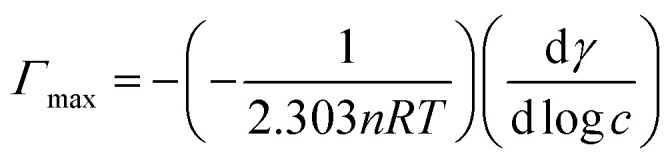
19
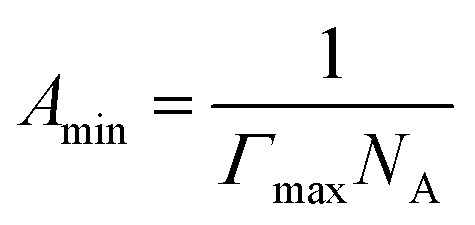


**Table 2:** Parameters of static surface tension

Sample	CMC (nmol L^−1^)	*γ*_cmc_ (mN m^−1^)	*Γ*_max_ (μmol m^−2^)	*A* (nm^2^)	PC_20_	Δ*G*_mic_ (KJ mol^−1^)	Δ*G*_ads_ (KJ mol^−1^)
TMA	2.376 ± 0.07	29.214 ± 0.15	1.54 ± 0.03	1.08 ± 0.02	4.3 ± 0.2	−25.36 ± 0.1	−25.64 ± 0.1
DMAE	0.857 ± 0.02	27.823 ± 0.1	1.37 ± 0.02	1.21 ± 0.02	5 ± 0.3	−27.08 ± 0.1	−27.41 ± 0.1
MDAE	0.539 ± 0.02	27.745 ± 0.1	0.90 ± 0.01	1.84 ± 0.03	5 ± 0.3	−27.08 ± 0.1	−27.58 ± 0.1

*T* represents the absolute temperature (298.15 K), *R* denotes the gas constant (8.314 J mol^−1^ K^−1^), and d*γ*/dlog *c* refers to the slope of the *γ*-log *c* curve below the critical micelle concentration (CMC). *n* is one constant determined by the number of species constituting the surfactant adsorbed at the interface. For linear cationic surfactants, *n* is equal to 2. *N*_A_ represents Avogadro's constant (6.022 × 1023).^15^

A key parameter for assessing surfactants is their efficiency in lowering surface tension, denoted as PC_20_. This value is determined using the formula provided below.^23^

The free energy values for micelle formation (Δ*G*_m_) and adsorption (Δ*G*_ads_) were determined through the application of eqn (4) and (5).^24^ The calculation results are shown in Table 2.

According to Table 2, the maximum adsorption capacity of the surfactant (*Γ*_max_) shows a decreasing trend with increasing number of hydroxyl groups, the minimum area per molecule (*A*_min_)increases. This phenomenon can be explained by the enhanced formation of hydrogen bonds among surfactant molecules as the hydroxyl group count rises, strengthening intermolecular interactions. Consequently, this reduces molecular mobility and adsorption capacity at the interface. Furthermore, the addition of hydroxyl groups increases the complexity of the surfactant's molecular structure, restricting the orderly arrangement of molecules at the interface. This steric hindrance effect can make it more difficult for the molecules to pack tightly, thereby reducing the maximum adsorption capacity (*Γ*_max_). At the same time, hydroxyl groups are hydrophilic groups, and as the number increases, the hydrophilicity of the surfactant molecules also increases. This causes the molecules to prefer to remain in the aqueous phase rather than adsorb at the interface, thereby reducing the adsorption capacity at the interface.

### 3.4 Dynamic surface tension

The migration of surfactant molecules from the liquid phase to the gas–liquid interface, followed by their re-adsorption at the interface, represents a dynamic equilibrium process in essence.^25^ Surfactant molecules gradually migrate to the air–liquid interface, replacing water molecules and weakening the cohesive forces at the interface, resulting in a reduction in surface tension.^26^ The adsorption equilibrium of surfactants on the solution surface usually occurs within a short time. The adsorption behavior of TMA, DMAE, and MDAE was studied using the maximum bubble pressure method through dynamic surface tension measurements.

#### 3.4.1 Rosen empirical formula

**Fig. 4:**
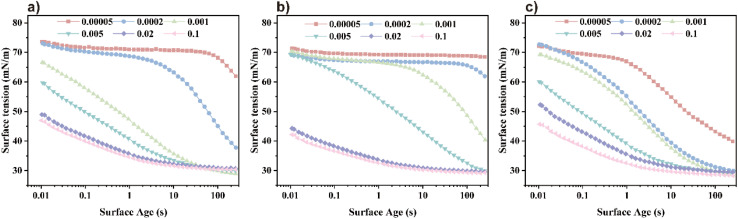
Dynamic surface tension at different concentrations: (a) TMA, (b) DMAE, and (c) MDAE.

Rosen *et al.* identified four distinct phases in the dynamic surface tension curve. These include the induction phase, during which surface tension exhibits minimal change, followed by the rapid decay phase, characterized by swift surfactant adsorption that significantly lowers surface tension. Subsequently, the intermediate equilibrium phase emerges, where the rate of adsorption decreases and surface tension approaches stability. Finally, the equilibrium phase is reached, marked by saturated adsorption and a constant surface tension.^26–28^ As depicted in Fig. 4, at lower concentrations, the induction phase and rapid decay stage prevail, with surfactant molecules exhibiting slower diffusion and adsorption. With increasing concentration, the induction phase shortens, allowing molecules to adsorb more rapidly onto the interface. The time to reach equilibrium also decreases significantly, reflecting the impact of concentration on the dynamic adsorption behavior. The Rosen *et al.* proposed empirical formula(6). Describes the temporal variation of the dynamic surface tension.^29–31^

**Fig. 5:**
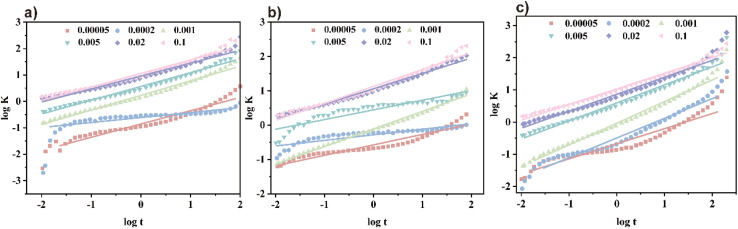
Calculation of dynamic surface tension parameters: (a) TMA, (b) DMAE, and (c) MDAE.

The fitted curve, illustrating surface tension, is presented in Fig. 5. The slope of the curve is denoted by *n*, while t* represents the intercept. Corresponding calculation outcomes are summarized in Table 3. Utilizing eqn (8) and (9), *t*_i_ and *t*_m_ are derived.^16^

**Table 3:** Dynamic surface tension parameters

Sample	Concentration (mol L^−1^)	*n*	t* (s)	log t*	*t*_i_ (s)	*t*_m_ (s)	*R*_1/2_ (mN m^−1^ s^−1^)
TMA	5 × 10^−5^	0.21	821.64	2.91	0.01	5.71 × 10^7^	0.01
	2 × 10^−4^	0.56	29.58	1.47	0.5	1.76 × 10^3^	0.58
	1 × 10^−3^	0.59	0.37	−0.43	0.01	17.98	57.82
	5 × 10^−3^	0.56	0.09	−1.06	0.001	5.34	241.79
	5 × 10^−2^	0.49	0.01	−1.96	0.0001	1.22	1873.38
	1 × 10^−1^	0.5	0.01	−2.11	7.43 × 10^−5^	0.81	2693.46
DMAE	5 × 10^−5^	0.05	87.01	−2.04	5.16 × 10^−7^	163.31	203.19
	2 × 10^−4^	0.15	58.97	1.78	7.68 × 10^−6^	4.53 × 10^8^	0.07
	1 × 10^−3^	0.38	23.15	1.36	0.057	9.36 × 10^3^	0.69
	5 × 10^−3^	0.57	1.32	0.12	0.024	73.04	16.03
	5 × 10^−2^	0.47	0.01	−2.17	5.24 × 10^−5^	0.87	3033.28
	1 × 10^−1^	0.49	0.002	−2.6	2.35 × 10^−5^	0.27	8121.38
MDAE	5 × 10^−5^	0.62	35.92	0.77	0.15	240.75	2.69
	2 × 10^−4^	0.49	22.43	1.35	0.21	2421.61	0.8
	1 × 10^−3^	0.67	1.13	0.05	0.037	34.31	18.85
	5 × 10^−3^	0.56	0.08	−1.08	0.001	5.22	257.63
	5 × 10^−2^	0.53	0.02	−1.64	0.0003	1.79	935.47
	1 × 10^−1^	0.49	0.01	−2.13	6.50 × 10^−5^	0.85	2953.06

Table 3 shows the calculation results of the above parameters. Here, *n* reflects the diffusion process of surfactant molecules from the bulk phase to the subsurface layer in the early stage, while t* reflects the adsorption process of surfactant molecules from the subsurface layer to the surface in the later stage. With increasing concentration, the *n* values of TMA, DMAE, and MDAE initially increase and then decrease. At elevated surfactant concentrations, the diffusion barrier diminishes, facilitating swift molecular movement during the early adsorption stage. The reduced *t*_i_ value provides additional confirmation of this phenomenon. With increasing concentration, t* declines, indicating a reinforcement of the barrier during the later stages of adsorption. This effect is primarily attributed to electrostatic repulsion and the decreased availability of adsorption sites. By comparing TMA, DMAE, and MDAE, it can be observed that at low concentrations, the differences in *n* are not significant. However, the t* values of TMA, DMAE, and MDAE clearly follow the order TMA > DMAE > MDAE. This may be due to the more pronounced hydrogen bonding interactions between surfactant molecules with more hydroxyl groups and solvent molecules (such as water). These strong intermolecular interactions increase the viscosity of the system and thereby slow the diffusion rate of the molecules. Furthermore, the strong polarity of the hydroxyl groups means that increasing their number increases the overall polarity of the surfactant molecules. This increases the interactions between the molecules and the polar solvent, making it more difficult for the molecules to migrate from the bulk solution to the gas–liquid interface, further slowing the rate of diffusion.

#### 3.4.2 Diffusion-controlled adsorption model

The adsorption kinetics model at the gas–liquid interface posits the existence of a thin layer beneath the interface, comprising only a few molecular layers. Before adsorption occurs, surfactant molecules typically must traverse this thin layer and reach the interface *via* a self-diffusion mechanism. When the adsorption process between the interface and the thin film reaches a local equilibrium state, the Ward–Tordai equation can be used to describe and analyze this process:^32^

Solving the Ward–Tordai equation is often challenging, particularly due to the complexity associated with measuring the dynamic adsorption capacity during the process. In the initial stage of adsorption (*i.e.*, when *t* → 0), reverse adsorption has not yet occurred. During this stage, the Ward–Tordai equation can be approximated using the Fickian diffusion equation, providing a more precise representation of the adsorption behavior in this phase.^33^

As *t* → ∞, the subsurface concentration approaches equilibrium with the surface concentration. For long-term adsorption, the Joos equation is employed as an approximation of the Ward–Tordai equation.^34^

**Fig. 6:**
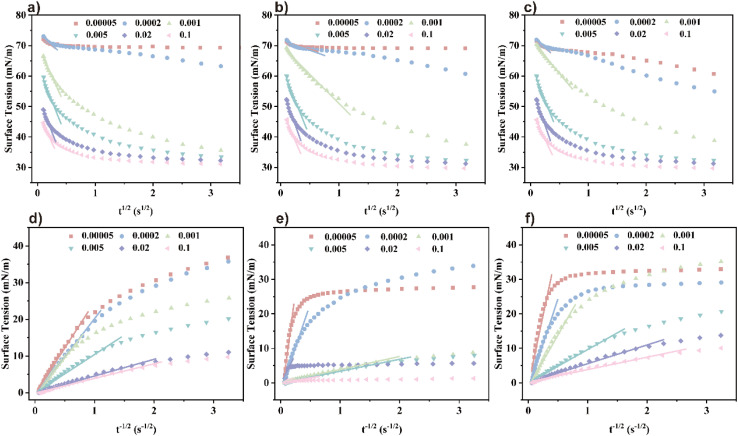
Diffusion coefficients calculated at different concentrations: (a)–(c) are TMA, DMAE, MDAE (*t* → 0), (d)–(f) are TMA, DMAE, MDAE (*t* → ∞) respectively.

As shown in Fig. 6, *γ*_*t*_ changes varies with *t*^±1/2^ and exhibits noticeable has clear inflection points, indicating suggesting that the adsorption process is not completely diffusion-controlled. Diffusion controlled is. The diffusion coefficient *D* at *t* → 0 and *t* → ∞ was determined by fitting the curve, with the curve. The results are shown in Table 4. From Table 4 and it can be observed that for the three surfactants at the same concentration, *D* → 0 is greater than *D* → ∞.

**Table 4:** Diffusion coefficient of TMA, DMAE and MDAE

Sample	*D*_*t*→0_ (m^2^ s^−1^)	*D*_*t*→∞_ (m^2^ s^−1^)	*D*_*t*→∞_/*D*_*t*→0_
TMA	3.93191 × 10^−12^	5.74645 × 10^−14^	0.014614916
DMAE	5.09992 × 10^−12^	9.33722 × 10^−14^	0.018308556
MDAE	7.50514 × 10^−12^	1.94261 × 10^−13^	0.002588368

In the early stages of the wetting process, intermolecular forces and adsorption barriers are minimal, allowing surfactant molecules to spread rapidly. Over time, surfactant molecules accumulate at the interface, potentially undergo back diffusion, and enter an expansion phase that slows the rate of diffusion of the remaining unabsorbed molecules.^35^

By comparing the diffusion coefficients, it is concluded that DTMA < DDMAE < DMDAE. This trend may be attributed to the increased number of hydroxyl groups, which could lead to the formation of more hydrogen bonds between surfactant molecules. These hydrogen bonds alter the polarity of the molecules, increasing their solubility in water. This enhanced solubility facilitates faster diffusion of the molecules from the bulk solution to the gas–liquid interface, thereby increasing the diffusion coefficient. At this point, *D* → 0/*D* → ∞< 1 indicates that the adsorption process involves a mixed diffusion-kinetic adsorption mechanism in which the initial diffusion occurs relatively quickly, while the subsequent adsorption rate is limited by kinetic factors.

### 3.5 Dynamic contact angle

To evaluate the wetting performance of surfactant solutions, the contact angle between the liquid and the solid surface is typically used as a key parameter. This angle is influenced by the interfacial tension between liquid and gas *γ*_gl_ and between liquid and solid *γ*_ls_,^36^ it can be calculated by eqn (14).

**Fig. 7:**
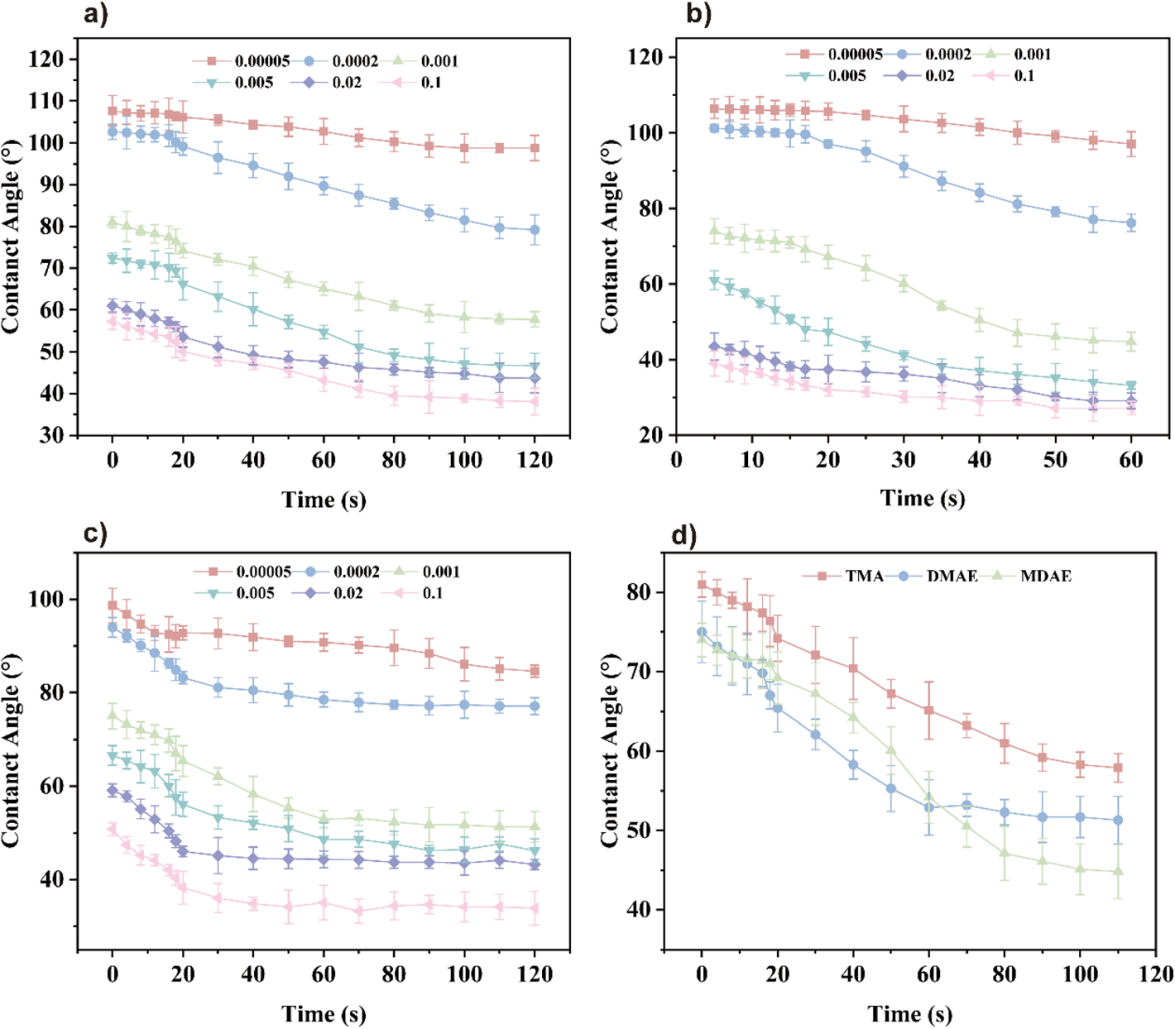
Dynamic contact angles: (a) contact angles of TMA at different concentrations, (b) contact angles of DMAE at different concentrations, (c) contact angles of MDAE at different concentrations, (d) comparison of contact angle properties of three surfactants at the same concentration.

**Fig. 8:**
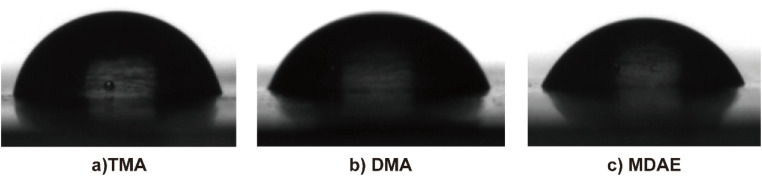
Photo of the contact angle of surfactant with a concentration of 0.001 mol L^−1^ on the paraffin film.

Interfacial tension, influenced by the surfactant type and concentration, directly impacts the contact angle (*θ*), making it a critical macroscopic marker of adsorption behavior at the interface. When the contact angle *θ* is less than 90°, the solution can normally wet the substrate, which is a commonly used criterion for evaluating wetting performance.^37^ The *θ* curves of surfactant solutions shown in Fig. 7a and c show a rapid decrease with increasing surface age within a short time, followed by a stabilization until an equilibrium state where *θ* is less than 90°. Fig. 7 illustrates the time-dependent dynamic contact angle behavior of the three surfactants at a concentration of 0.001 mol L^−1^, highlighting the evolving nature of their wetting process. The *θ* curve of the surfactant solution shown in Fig. 7a and c shows that the data is obtained through multiple tests (such as the five tests you mentioned) and the statistical error is evaluated by calculating the standard deviation or confidence interval. In a short period of time, it declines rapidly with the increase of surface age, and then stabilizes until the equilibrium state of *θ* is less than 90°. Fig. 7 illustrates the time-related dynamic contact angle behavior of three surfactants at a concentration of 0.001 mol L^−1^, highlighting the evolutionary properties of their wetting process. Fig. 8 shows photos of the contact angle at 0 seconds, illustrating the differences in initial wettability. Once the surfactant concentration exceeds the critical micelle concentration (CMC), all contact angles drop below 90°, indicating a substantial improvement in wettability. From the figure it can be seen that under the condition of the same concentration of three surfactants, the order of the contact angle is: MDAE > DMAE > TMA. This may be due to the fact that MDAE's surfactant is more evenly distributed on the liquid surface, but its strong hydrophilicity makes the molecules more prone to integrate into the aqueous phase, resulting in a reduction in the aggregation of molecules at the solid–liquid interface, which increases the contact angle.

### 3.6 Application performance

#### 3.6.1 Foaming

Foaming ability consists of two main components: foamability and foam stability.^38,39^ Foam stability (IB) and foaming capacity (FC) are determined using the corresponding formulas (15) and (16).^40,41^

**Fig. 9:**
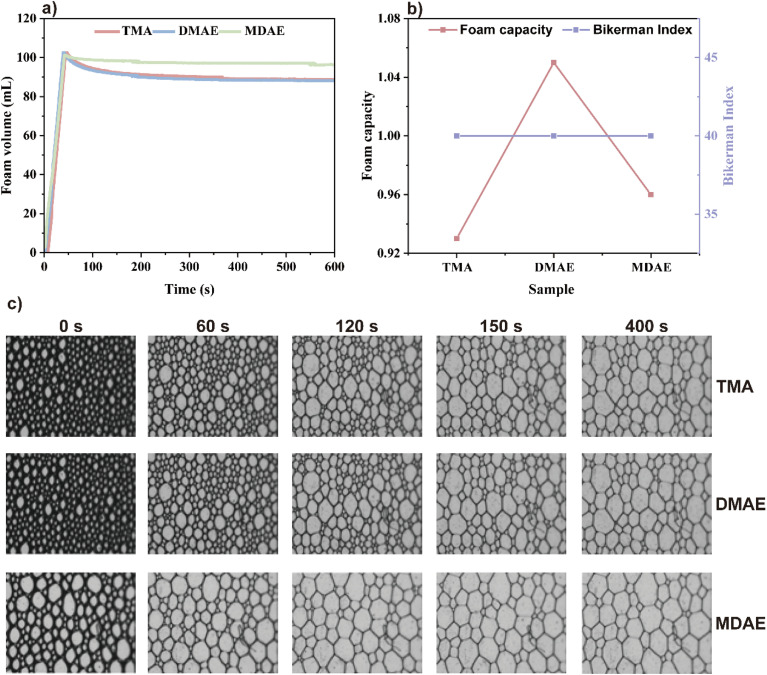
(a) Time-dependent changes in the foam volume of the three surfactants at the same concentration. (b) Comparison of the foaming ability and foam stability of the three surfactants, (c) CCD images showing the time-dependent changes in foam structure of TMA, DMAE, and MDAE at 25 °C.

The Bikerman index characterizes the stability of the foam volume (mL) produced by a given gas flow rate (mL min^−1^) and is represented as IB. Its calculation formula is:

Fig. 9a and b show the foaming performance of the three surfactants. From the curves in Fig. 9a, it can be seen that the foam volume increases with time during the aeration period. Once aeration stops and drainage occurs, the foam in the solution begins to break down. From Fig. 9b, it can be seen that the foamability (FC) of the three surfactants follows the order: DMAE > MDAE > TMA. This may be because the molecular structure of DMAE with two hydroxyl groups is likely to achieve an optimal balance between hydrophobic tail and hydrophilic head, resulting in higher adsorption efficiency at the gas–liquid interface. Molecules with a single hydroxyl group may exhibit slightly greater hydrophobicity, resulting in a less compact alignment at the interface. On the other hand, molecules with three hydroxyl groups are excessively hydrophilic, resulting in insufficient adsorption capacity at the gas–liquid interface. From the images captured by the CCD camera in Fig. 9c, it can be seen that the foam of the three surfactants continuously accumulates over time. In addition, the foam of all three surfactants remains stable within a certain period of time.

#### 3.6.2 Salt and alkali tolerance

**Fig. 10:**
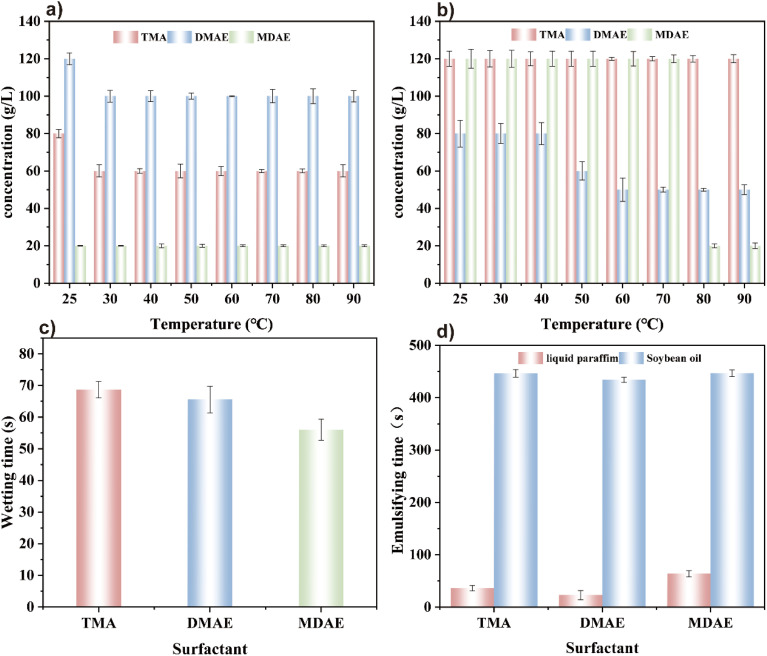
Shows the salt and alkali resistance performance of the three surfactants under differenttemperatures and concentrations: (a) salt resistance performance, (b) alkali resistance performance, (c) wetting performance of TMA, DMAE, and MDAE at the same concentration, (d) emulsification performance of TMA, DMAE, and MDAE at the same concentration.

From Fig. 10a, the concentration of salt and alkali is usually calculated by precision balance and solubility. The error is estimated by the standard error of concentration. It can be concluded that DMAE has relatively better salt tolerance at a certain temperature and salt concentration. In contrast, the salt tolerance of TMA and MDAE gradually deteriorates with increasing temperature and salt concentration, while the salt tolerance of DMAE is the worst. When the number of hydroxyl groups is moderate (for example, two hydroxyl groups), the molecule exhibits better hydration and dispersion stability in a high salt environment. However, when the number of hydroxyl groups is too low (for example, one hydroxyl group) or too high (for example, three hydroxyl groups), the molecular stability in the physiological saline solution is reduced.

From Fig. 10b, it can be concluded that TMA has excellent alkali resistance at a certain temperature and alkali concentration. In contrast, MDAE's alkali resistance drops sharply at 80 °C, and DMAE's alkali resistance is also affected by temperature and its alkali resistance is worse than that of the other two. This may be because molecules with a single hydroxyl group (TMA) have a lower probability of forming alkoxides under alkaline conditions, resulting in higher overall stability of the molecule. In contrast, molecules with two hydroxyl groups (DMAE) can simultaneously transform into alkoxides in an alkaline environment, significantly increasing molecular negativity. This shift disrupts the hydrophilic/hydrophobic balance of the surfactant, resulting in a decrease in performance. Although MDAE has more hydroxyl groups, the molecules can form stable structures through intermolecular or intramolecular hydrogen bonds, which mitigates the destructive effects of the alkaline environment, thereby exhibiting better alkali resistance compared to surfactants with two hydroxyl groups.

#### 3.6.3 Wettability

Wettability is assessed by replacing the gas–solid interface with a liquid–solid interface and determining the wetting time on a standard canvas. Shorter wetting times indicate enhanced wettability, as the liquid spreads more rapidly and makes contact with the surface more effectively. As shown in Fig. 10c, quantify the random error through statistical methods (standard deviation and RSD), and estimate the overall error in combination with the system error (deviation), the error of each measurement is determined, showing the wettability test results of TMA, DMAE and MDAE with a concentration of 10 g L^−1^. It can be seen from the figure that the wetting time of the canvas net in the surfactant solution is relatively short. This is because surfactants have lower surface tension and allow for quick wetting of the canvas. Furthermore, the wetting time follows the order TMA > DMAE > MDAE, which may be due to the increasing number of hydroxyl groups in the molecule. This increases the overall hydrophilicity of the surfactant molecules, making them more easily dissolved or dispersed in the aqueous phase, thereby speeding up the wetting process. The polarity effect of hydroxyl groups decreases the interfacial tension between the liquid and the solid surface. This reduction facilitates quicker liquid spreading across the solid surface and minimizes the time required for wetting.

#### 3.6.4 Emulsifying power

The emulsification performance of TMA, DMAE and MDAE at the same concentration was tested using liquid paraffin and soybean oil as oil phases. The results are shown in Fig. 10d, quantify the random error through statistical methods (standard deviation and RSD), and estimate the overall error in combination with the system error (deviation), it can be seen that the emulsification ability of the three different surfactants in soybean oil and liquid paraffin is essentially the same, indicating that the variation in the number of hydroxyl groups has a negligible effect on the emulsification performance.

#### 3.6.5 Anti-static power

Antistatic performance is determined by the material's ability to dissipate surface charges, which can be evaluated by measuring surface resistance (*R*_s_) to assess the electrostatic degradation rate. Based on this measurement, the performance of TMA, DMAE and MDAE is compared and evaluated. Rs can be measured with a high-resistance meter, with the concentration of the test sample being 10 g L^−1^. The surface resistance (*ρ*_s_) can be calculated using the following formula(17).

To evaluate the antistatic properties of surfactants, the logarithmic reduction in surface resistance (Δlog *R*_s_) of treated polyester fabric is used as a key metric. A greater Δlog *R*_s_ indicates superior antistatic efficacy. The calculation results are shown in Table 5. The error of *ρ*_s_ mainly comes from the uncertainty of *R*_s_, and the logarithmic transformation error conforms to the error propagation theory. According to the test results, DMAE achieves the highest Δlog *R*_s_ value of 4.91 Ω, demonstrating the most effective antistatic performance. This is possibly because the two hydroxyl groups provide optimal balance and allow the molecules to form a stable adsorption layer on the surface, effectively reducing static charge accumulation. In contrast, surfactants with a single hydroxyl group may lack sufficient hydrogen bonding interactions, while surfactants with three hydroxyl groups may experience excessively strong intermolecular attraction due to excessive hydrogen bonds, which could destroy the surface structure and compromise antistatic performance.

**Table 5:** The surface resistance of polyester fabrics subjected to treatment with TMA, DMAE, and MDAE was analyzed

Sample	*R*_s_ (Ω)	*ρ*_s_ (Ω)	log*R*_s_ (Ω)	Δlog*R*_s_ (Ω)
TMA	(1.76 ± 0.03) × 10^10^	(1.36 ± 0.03) × 10^11^	10.25 ± 0.02	3.29 ± 0.02
DMAE	(4.26 ± 0.04) × 10^8^	(3.28 ± 0.04) × 10^9^	8.63 ± 0.01	4.91 ± 0.01
MDAE	(3.46 ± 0.01) × 10^9^	(2.66 ± 0.01) × 10^10^	9.54 ± 0.03	4.00 ± 0.03

## 4. Conclusion

In this study, three cationic surfactants containing varying numbers of hydroxyl groups were synthesized and assessed, with the impact of hydroxyl group quantity on their properties thoroughly examined. The research results show that FTIR, ^1^H NMR and thermal performance analysis prove that the target product is generated and the increase in the number of hydroxyl groups leads to the improvement of thermal stability, and the increase in the number of hydroxyl groups will significantly change the molecular configuration of the surfactant and its interface properties. The diffusion coefficient increases with increasing number of hydroxyl groups, suggesting that polyhydroxyl surfactants (such as MDAE) have a faster molecular migration rate and interfacial coverage under dynamic interfacial conditions. This performance increase is particularly important when applying short-term rapid surface tension reduction. With regard to the specific application performance, cationic surfactants with different hydroxyl groups show clear differences. Monohydroxy surfactants are suitable for scenarios with high requirements for alkali resistance and static adsorption; dihydroxy surfactants are suitable for high salinity environments and foaming applications with excellent overall performance; polyhydroxy surfactants. It is suitable for applications with dynamic interface conditions and significant wetting requirements. In summary, the study demonstrated that the quantity of hydroxyl groups governs the functionality of cationic surfactants.

## Data availability

The data used in this study come from publicly accessible databases and data generated through experiments. Public datasets can be accessed through major databases. However, due to ethical and privacy restrictions, some experimentally generated data are not suitable for direct public release. To ensure the reproducibility of the study and the transparency of the results, we are willing to provide necessary data support upon reasonable request.

## Author contributions

All the authors have accepted responsibility for the entire content of this submitted manuscript and approved submission.

## Conflicts of interest

The authors declare no conflicts of interest regarding this article.

## References

[cit1] Kalam S., Abu-Khamsin S. A., Patil S., Hussain S. M. S., Mahmoud M., Kamal M. S., Shalabi E. W. A. (2023). Geoenergy Sci. Eng..

[cit2] Huang X., Sun L., Cao S., Xie W., Huo Y., Liu X., Xia J. (2024). J. Mol. Liq..

[cit3] Shahrashoob Z., Yu G., Long S., Grady B. P., Harwell J., Arhancet G. (2018). J. Surfactants Deterg..

[cit4] Kustov A. V., Krestyaninov M. A., Kruchin S. O., Shukhto O. V., Kustova T. V., Belykh D. V., Khudyaeva I. S., Koifman M. O., Razgovorov P. B., Berezin D. B. (2021). Mendeleev Commun..

[cit5] Phaodee P., Sabatini D. (2022). J. Am. Oil Chem. Soc..

[cit6] Chang H., Wang Y., Cui Y., Li G., Zhang B., Zhao X., Wei W. (2016). Colloids Surf., A.

[cit7] Phaodee P., Sabatini D. A. (2021). J. Surfactants Deterg..

[cit8] Sun Z., Ji Y., Wang H., Zhang J., Yuan C., Kang M., Feng Y., Yin H. (2024). Colloids Surf., A.

[cit9] Xiao X., Qiao Y., Xu Z. R., Wu T. Y., Wu Y. X., Ling Z., Yan Y., Huang J. B. (2021). Langmuir.

[cit10] Liu W. B., Liu W. G., Zhao Q., Shen Y. B., Wang X. Y., Wang B. Y., Peng X. Y. (2020). J. Mol. Liq..

[cit11] Zhang Z., Wang H., Shen W. (2013). J. Chem. Eng. Data.

[cit12] Li J., Li Y. L., Song Y. B., Wang Z. F., Zhang Q. H. (2018). J. Mol. Liq..

[cit13] Zhang Y., Luo Y., Wang Y., Zhang J., Feng Y. (2013). Colloids Surf., A.

[cit14] Chen J., Hu X. Y., Fang Y., Xia Y. M. (2020). J. Mol. Liq..

[cit15] Phares R. E. (1965). J. Pharm. Sci..

[cit16] Matsubara H., Xu X. L., Bochi A., Aratono M. (2023). J. Oleo Sci..

[cit17] Chattoraj D. K. and Birdi K. S., Adsorption and the Gibbs surface excess, Plenum Press, 1984

[cit18] Pal N., Saxena N., Laxmi K. V. D., Mandal A. (2018). Chem. Eng. Sci..

[cit19] Aziz H. A., Yusoff R., Cheng N. G., Idris Z., Ramli N. A. S. (2023). Tenside, Surfactants, Deterg..

[cit20] Saito K., Onizawa Y., Kusama H., Iwasawa N. (2010). Chem.–Eur. J..

[cit21] Lyubimov S. E., Zvinchuk A. A., Chowdhury B., Davankov V. A. (2020). Russ. Chem. Bull..

[cit22] Feng N., Zhao T., Zhao Y. H., Song P., Li G. J., Zhang G. L. (2020). Colloids Surf., A.

[cit23] Li G., Liu X., Niu J. (2022). Appl. Chem. Ind..

[cit24] Li P. H., Cao S. T., Huo Y. Q., Liu X. C. (2024). J. Dispersion Sci. Technol..

[cit25] Gao T., Rosen M. J. (1995). J. Colloid Interface Sci..

[cit26] Ding H., Jiang Y. J., Wang Y. K., Ju H. B., Geng T. (2021). J. Mol. Liq..

[cit27] Hua X. Y., Rosen M. J. (1988). J. Colloid Interface Sci..

[cit28] Xi Y. H., Rosen M. J. (1991). J. Colloid Interface Sci..

[cit29] Chen Y., Xu G. (2013). Colloids Surf., A.

[cit30] Xingang W., Xiaoyi Y., Yongqiang S., Chaohua G., Ping L., Jianbo L. (2020). Tenside, Surfactants, Deterg..

[cit31] Wang W., Li J., Yang X., Li P., Guo C., Li Q. (2015). J. Mol. Liq..

[cit32] Varadaraj R., Bock J., Valint P., Zushma S., Brons N. (1990). J. Colloid Interface Sci..

[cit33] Fainerman V. B., Makievski A. V., Miller R. (1994). Colloids Surf., A.

[cit34] Rillaerts E., Joos P. (1982). J. Phys. Chem..

[cit35] Fan J. M., Zhang J., Yang X. Q., Bai L., Zhou Y., Wu Z. Y., Qin Z. Y. (2022). Colloids Surf., A.

[cit36] Zhang J. G., Liu H. H., Ba Y. (2019). Langmuir.

[cit37] Krussmann H., Bercovici R. (1991). J. Chem. Technol. Biotechnol..

[cit38] Lioumbas J. S., Georgiou E., Kostoglou M., Karapantsios T. D. (2015). Colloids Surf., A.

[cit39] Acharya D. P., Gutiérrez J. M., Aramaki K., Aratani K., Kunieda H. (2005). J. Colloid Interface Sci..

[cit40] Zhou Y. W., Wang S., Lv M. D., Niu J. P., Xu B. C. (2017). J. Surfactants Deterg..

[cit41] Wang Y., Liu X. C., Zhou Y. W., Niu J. P. (2016). J. Surfactants Deterg..

